# Regulation of the actin cytoskeleton by the Ndel1-Tara complex is critical for cell migration

**DOI:** 10.1038/srep31827

**Published:** 2016-08-22

**Authors:** Ji-Ho Hong, Yongdo Kwak, Youngsik Woo, Cana Park, Seol-Ae Lee, Haeryun Lee, Sung Jin Park, Yeongjun Suh, Bo Kyoung Suh, Bon Seong Goo, Dong Jin Mun, Kamon Sanada, Minh Dang Nguyen, Sang Ki Park

**Affiliations:** 1 Department of Life Sciences, Pohang University of Science and Technology, Pohang 790-784, Republic of Korea; 2 Molecular Genetics Research Laboratory, University of Tokyo, 7-3-1 Hongo, Bunkyo-ku, Tokyo 113-0033, Japan; 3 Hotchkiss Brain Institute, Departments of Clinical Neurosciences, Cell Biology and Anatomy, and Biochemistry and Molecular Biology, University of Calgary, Calgary T2N 4N1, Canada

## Abstract

Nuclear distribution element-like 1 (Ndel1) plays pivotal roles in diverse biological processes and is implicated in the pathogenesis of multiple neurodevelopmental disorders. Ndel1 function by regulating microtubules and intermediate filaments; however, its functional link with the actin cytoskeleton is largely unknown. Here, we show that Ndel1 interacts with TRIO-associated repeat on actin (Tara), an actin-bundling protein, to regulate cell movement. *In vitro* wound healing and Boyden chamber assays revealed that Ndel1- or Tara-deficient cells were defective in cell migration. Moreover, Tara overexpression induced the accumulation of Ndel1 at the cell periphery and resulted in prominent co-localization with F-actin. This redistribution of Ndel1 was abolished by deletion of the Ndel1-interacting domain of Tara, suggesting that the altered peripheral localization of Ndel1 requires a physical interaction with Tara. Furthermore, co-expression of Ndel1 and Tara in SH-SY5Y cells caused a synergistic increase in F-actin levels and filopodia formation, suggesting that Tara facilitates cell movement by sequestering Ndel1 at peripheral structures to regulate actin remodeling. Thus, we demonstrated that Ndel1 interacts with Tara to regulate cell movement. These findings reveal a novel role of the Ndel1-Tara complex in actin reorganization during cell movement.

Cell motility is central to many biological processes such as embryonic development, tissue repair, immune responses, and cancer metastasis^1^. Motility requires the precisely integrated regulation of various cellular processes, including dynamic cytoskeletal remodeling^2^,^3^,^4^,^5^. Reorganization of actin filaments is controlled by actin-associated proteins that control nucleation, branching, severing, bundling, elongation, and capping^4^,^5^,^6^,^7^,^8^. Orchestrated regulation of these actin polymerization factors leads to distinct changes in actin cytoskeleton architecture^5^,^6^,^7^, thereby regulating cellular processes that impact mitosis, cytokinesis, endocytosis, and cell migration^3^,^7^,^8^,^9^,^10^. In cancer metastasis, actin-dependent protrusion of cell pseudopodia is a critical element of mesenchymal cell migration driven by cycles of actin polymerization^11^,^12^,^13^. Consistent with these findings, a considerable number of studies have demonstrated that multiple actin-associated proteins are involved in the enhanced movement of tumor cells^11^,^12^,^13^,^14^,^15^.

Nuclear distribution element-like 1 (Ndel1), a 345 amino acids coiled-coil domain-containing protein, is the mammalian homolog of *Aspergillus nidulans* NudE, which was originally identified as a factor that regulates molecular motors in various cell types^16^,^17^,^18^,^19^. Ndel1 ensures the assembly of the mitotic spindle, centrosomal maturation, and mitosis through its association with microtubules prior to mitotic entry at the G2/M phase^20^,^21^,^22^,^23^. The function of Ndel1 in the brain has been extensively investigated^17^,^18^,^24^,^25^,^26^,^27^,^28^,^29^. Studies have shown that it induces neuronal differentiation and maintains the integrity of maturing neurons through polymerization of neurofilaments transported by dynein and kinesin^24^. In association with dynein and Lis1, Ndel1 contributes to neuronal migration in the developing neocortex by stabilizing microtubules and promoting nucleokinesis^25^. Recent evidence also hints at the involvement of Ndel1 in tumorigenesis and carcinogenesis^30^,^31^. Collectively, the current understanding of Ndel1 function is mostly associated with microtubule dynamics, whereas knowledge of its link to actin filaments is limited^19^,^32^,^33^,^34^.

TRIO binding protein-1 (TRIOBP-1), also known as TRIO-associated repeat on actin (Tara), is a filamentous actin (F-actin)-binding protein that was originally identified as a TRIO-associated factor. TRIO, a member of the Rho guanine nucleotide exchange factor family that can exchange guanine nucleotides on Rho GTPase^35^,^36^,^37^, is important for regulating actin filament reorganization, cell motility, cell proliferation, and axonal development^38^,^39^,^40^. As an interacting partner of TRIO, TRIOBP-1 is linked to actin cytoskeleton organization, and a deficiency of this protein causes embryonic lethality in mice^35^,^41^. The *TRIOBP* gene encodes multiple splice variants that generate three major forms of the protein, namely, TRIOBP-5 (long isoform of approximately 2,300 amino acids), TRIOBP-4 (mainly the N-terminus of TRIOBP-5), and TRIOBP-1 (mainly the C-terminus of TRIOBP-5, also called Tara)^42^,^43^. Tara consists of an N-terminal pleckstrin homology (PH) domain and a C-terminal coiled-coil region, the latter of which is responsible for homodimerization^35^,^44^. TRIOBP-1 is expressed in most tissues, including those of the nervous system, while the other isoforms are expressed in a more limited range of tissues, such as the retina and inner ear^42^,^43^. To date, most studies of TRIOBP have focused on the relationships between TRIOBP-4/5 and hearing impairments^41^,^42^,^43^, whereas the biological function of Tara (TRIOBP-1) remains less clear.

Here, we investigated the functions of Ndel1 and Tara in cell movement. Our results reveal that Tara forms a functional complex with Ndel1 and alters its intracellular distribution. We also demonstrate that the Ndel1-Tara complex plays a role in regulating actin cytoskeleton organization, which is critical for cell migration.

## Results

### Tara interacts directly with Ndel1

**Figure 1:**
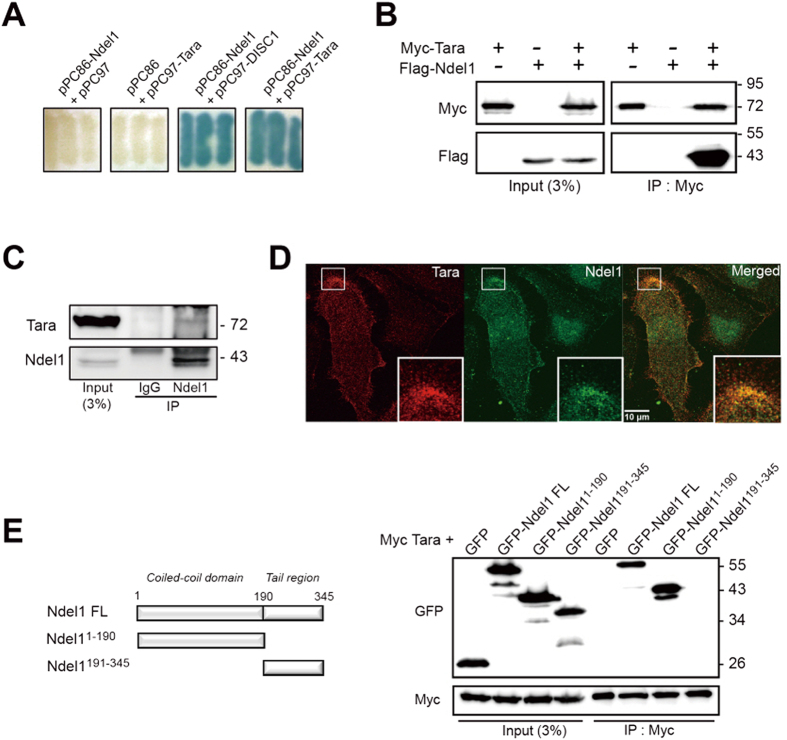
Tara interacts with Ndel1. (**A**) Interactions of Tara and Ndel1 in a yeast two-hybrid assay. Interaction-dependent β-galactosidase expression of co-transformants with the indicated constructs are shown. Co-transfection with pPC97-DISC1 and pPC86-Ndel1 was used as a positive control. (**B**) Co-immunoprecipitation of Ndel1 with full-length Tara. Flag-Ndel1 and Myc-Tara were transiently transfected into HEK293 cells and lysates were immunoprecipitated with anti-Myc. Immunoprecipitates were analyzed by immunoblotting with anti-Flag and anti-Myc. IP, immunoprecipitation. (**C**) Co-immunoprecipitation of endogenous Tara and Ndel1 from HEK293 cell lysates. Anti-Ndel1 immunoprecipitates were analyzed by immunoblotting with anti-Ndel1 and anti-Tara antibodies. (**D**) Ndel1 co-localizes with Tara at the peripheral region of SH-SY5Y cells. Endogenous Tara and Ndel1 were stained with anti-Tara (red) and anti-Ndel1 antibody (green), respectively. (**E**) HEK293 cell lysates expressing Myc-Tara and GFP-tagged fragments of Ndel1 were immunoprecipitated with anti-Myc antibody and immunoblots were probed with anti-Myc and anti-GFP.

In a yeast two-hybrid screen using human Ndel1 as bait, Tara was identified as a positive clone (Fig. 1A); hence, we obtained a full-length cDNA clone of Tara by RT-PCR using total RNA isolated from HEK293 cells as a template. We also confirmed that co-transformants containing Ndel1 and Tara showed interaction-dependent β-galactosidase activity (Fig. 1A). DISC1 and Trio, Ndel1- and Tara-interacting proteins, were used as positive controls for the assay (Figs 1A and S1A). Human Tara is a 593 amino acids protein (predicted molecular weight of 70 kDa) that harbors a PH domain at the N-terminus and a coiled-coil domain at the C-terminus. To determine whether Ndel1 interacts with Tara in mammalian systems, we performed a co-immunoprecipitation assay using HEK293 cells expressing Flag-Ndel1 and Myc-Tara, and observed a positive biochemical interaction, indicating the formation of a protein complex (Figs 1B and S1B). Consistently, endogenous Tara and Ndel1 were co-immunoprecipitated from HEK293 and SH-SY5Y cell lysates (Figs 1C and S1C,D). Immunocytochemical analyses using SH-SY5Y cells revealed extensive co-localization of Tara and Ndel1 at the periphery, providing evidence for complex formation between these proteins (Figs 1D and S1E–G). Notably, co-localization of endogenous Ndel1 and Tara was enriched at the leading edge of SH-SY5Y cells (Fig. S1E), suggesting the potential involvement of the Ndel1-Tara complex in cell movement. Since Tara is a binding partner of Trio (Fig. S1H, a) and Ndel1 binds to DISC1 (Fig. S1H, b) and Lis1^17^,^27^,^35^, we performed co-immunoprecipitation assays and examined whether the interaction between Tara and Ndel1 affected interactions among the other interaction partners. When Tara was co-expressed, there were no significant changes in the interactions between Ndel1 and Disc1 (Fig. S1I) or Ndel1 and Lis1 (Fig. S1J), which was also the case for Ndel1 in the interaction between Tara and Trio^*1118*–*1919*^, which is known as the binding region of Trio for Tara^35^ (Fig. S1K). To characterize the interaction between Tara and Ndel1 further, we aimed to determine the regions of Ndel1 responsible for complex formation. A series of Ndel1 truncation mutants were generated based on domain predictions, and their interactions with Tara were assayed by co-immunoprecipitation using HEK293 cell lysates. The N-terminal coiled-coil domain of Ndel1 (amino acids 1–190; Ndel1^1–190^) co-immunoprecipitated with full-length Tara, whereas the C-terminal tail region of Ndel1 (amino acids 191–345; Ndel1^191–345^) did not (Fig. 1E), indicating that the coiled-coil region of Ndel1 is mainly involved in the interaction with Tara. We also examined whether Tara and Ndel1 interacted directly using a blot overlay assay. When purified recombinant GST-Tara was separated by SDS-PAGE, transferred to PVDF membrane followed by overlay with purified recombinant His-Ndel1, His-Ndel1 was detected at a molecular weight corresponding to that of GST-Tara (Fig. S1L), indicating their direct interaction.

### Tara and Ndel1 regulate cell migration

**Figure 2:**
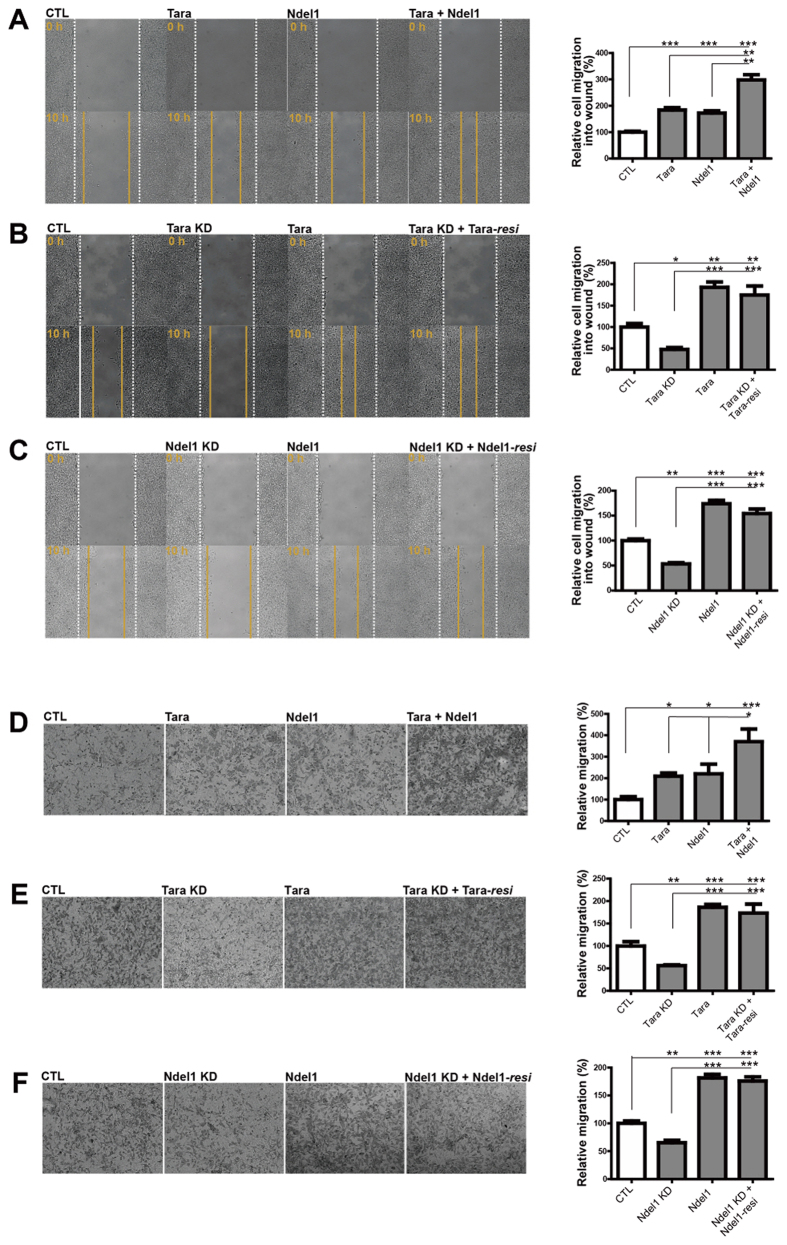
Ndel1 and Tara regulate cell migration. (**A**–**C**) The effect of Ndel1 and Tara on wound healing of SH-SY5Y cells. Representative images of SH-SY5Y cells tanked at 0 and 10 hours during scratch wound-healing assay. Dashed lines indicate the initial boundaries of the scratches (white lines, 0 h) and cell movement leading edges at subsequent time point (yellow lines). The specificity of the Tara or Ndel1 knockdown phenotypes were tested by restoration of the wild-type phenotype upon co-expression of an RNAi-resistant form of Tara (Tara-*resi*) or Ndel1 (Ndel1-*resi*). Representative figures of living SH-SY5Y cells transfected with the indicated constructs were analyzed at at least 24 hours after transfection. Over 200 cells were counted for each cell group. All results are expressed as mean ± SEM from at least three independent experiments. *p < 0.05, **p < 0.01, ***p < 0.001 by one-way ANOVA with Tukey’s multiple comparison test. (**D**–**F**) Evaluation of the effect of Ndel1 and Tara on SH-SY5Y cell migration by Boyden chamber transmigration assay. After at least 24 hours of transfection, SH-SY5Y cells transfected with the indicated constructs were seeded in the upper chamber of transwell plates. After 12 hours of incubation, cells that migrated to the lower surface of the filters were fixed, stained with cresyl violet and quantified. Three randomly selected fields were counted per each filter. The specificity of the Tara or Ndel1 knockdown phenotypes were tested by restoration of the wild-type phenotype upon co-expression of an RNAi-resistant form of Tara (Tara-*resi*) or Ndel1 (Ndel1-*resi*). Over 200 cells were counted for each cell group. All results are expressed as mean ± SEM from at least three independent experiments. *p < 0.05, **p < 0.01, ***p < 0.001 by one-way ANOVA with Tukey’s multiple comparison test.

Because Ndel1 is an important regulator of neuronal and non-neuronal cell migration, we hypothesized that Tara may also be associated with cellular movement. To test this hypothesis, we employed a wound healing assay in which a scratch wound was created in a monolayer of SH-SY5Y cells and the migration rate of cells into the scratch was monitored. Compared with control cells, those overexpressing Ndel1 or Tara exhibited a significant increase in cell migration at 10 h after scratching (Fig. 2A), indicating that both Tara and Ndel1 are functionally associated with the cell migration process. Notably, when Tara and Ndel1 were co-expressed, wound closure was remarkably accelerated (Fig. 2A), supporting the concept that these proteins collaborate to regulate cell migration. Consistently, wound closure was decreased significantly when Ndel1 or Tara was depleted by shRNA-mediated knockdown (Fig. 2B,C). In addition, shRNA constructs against the human form of Tara (Fig. S2A) and Ndel1 (Fig. S2B) along with the RNAi-resistant human Tara (Fig. S2C) and Ndel1 (Fig. S2D) were generated and utilized for further assays using SH-SY5Y cells. The specificity of the Tara and Ndel1 knockdown phenotypes were verified by restoration of the phenotype upon co-expression of RNAi-resistant forms of Tara or Ndel1, Tara-*resi* and Ndel1-*resi*, respectively (Fig. 2B,C). Notably, the delayed wound healing phenotype caused by Ndel1 knockdown was reversed by Tara overexpression (Fig. S2E). Overexpression of Ndel1 also reversed the Tara knockdown effect, but was not as effective as Tara overexpression in Ndel1 knockdown cells (Fig. S2F), indicating that Ndel1 and Tara likely function in an overlapping pathway but may have separate functions involved in wound healing. In addition, we also employed actinomycin D (1 μM) in the assay using SH-SY5Y cells to examine the potential contribution of cell proliferation^45^, and observed that the potential complication by differential cell proliferation was not prominent (Fig. S3A). Taken together, these results suggest the involvement of Ndel1 and Tara in cell migration.

To evaluate the roles of Ndel1 and Tara in cell migration, a Boyden chamber transmigration assay was used to analyze the motility of SH-SY5Y cells. In this experiment, the migrational abilities of cells overexpressing Ndel1 or Tara were dramatically increased when compared to that of control cells (Fig. 2D). Moreover, knockdown of Ndel1 or Tara caused a significant reduction of cell migration (Fig. 2E,F). Effective reversal of the knockdown phenotype by co-transfection of either the Tara-*resi* or Ndel1-*resi* constructs was also observed (Fig. 2E,F). Overall, the two independent assays of cell motility produced strikingly similar results, confirming the functions of Ndel1 and Tara in cellular migration.

### The Ndel1-Tara complex is critical for cellular movement

**Figure 3:**
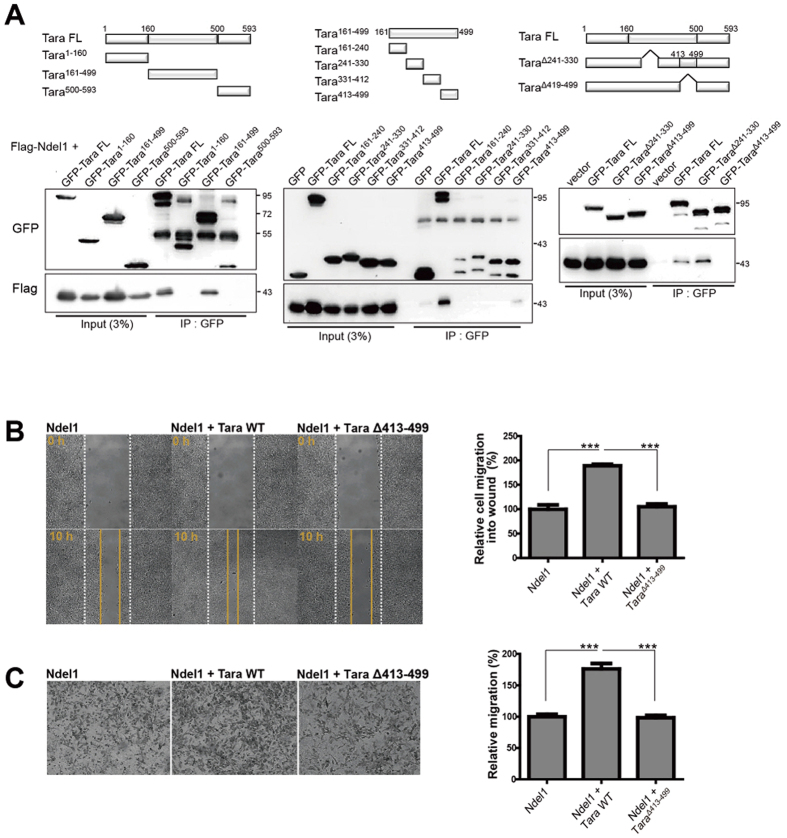
Direct interaction between Tara and Ndel1 is required for cell migration. (**A**) Domain organization of Tara and its fragments used for interaction domain mapping. Schematic diagram of the constructs used to map the Ndel1-Tara interaction is shown. (*left*) Ndel1 specifically interacts with 160–499 residues of Tara. HEK293 cell lysates expressing Flag-Ndel1 and GFP-tagged fragments of Tara were immunoprecipitated with anti-GFP antibody and immunoblots were probed with anti-Flag and anti-GFP. (*middle*) Tara fragment which consists of amino acid residues 160–499 of Tara was further divided into four pieces and paired as indicated. Flag-Ndel1 was co-expressed with various GFP tagged fragments of Tara in HEK293 cells. Immunoprecipitation was performed with anti-GFP antibody and immunoblotting was carried out with anti-Flag and anti-GFP. (*right*) Amino acid residues 413–499 of Tara are required for Ndel1 binding. HEK293 cell lysates expressing Flag-Ndel1 and GFP-tagged deletion mutants of Tara (Tara^Δ241–330^ and Tara^Δ413–499^) were immunoprecipitated with anti-GFP IP, and immunoblots were probed with anti-Flag and anti-GFP. Co-transfection with GFP-Tara^Δ241–330^ and Flag-Ndel1 was used as a positive control. (**B**) Representative images of SH-SY5Y cells tanked at 0 and 10 hours during scratch wound-healing assay. Dashed lines indicate the initial boundaries of the scratches (white lines, 0 h) and cell movement leading edges at subsequent time point (yellow lines). Over 200 cells were counted for each group. (**C**) Transwell migration assay using Boyden chamber was performed to examine the effect of Ndel1-Tara interaction on cell migration. After 24 hours of transfection, SH-SY5Y cells transfected with the indicated constructs were seeded in the upper chamber of transwell plates. After 12 hours of incubation, cells that migrated to the lower surface of the filters were fixed, stained with cresyl violet and quantified. Three randomly selected fields were counted per each filter. Over 200 cells were counted for each cell group. All results are expressed as mean ± SEM from at least three independent experiments. *p < 0.05, **p < 0.01, ***p < 0.001 by one-way ANOVA with Tukey’s multiple comparison test.

To verify the importance of Ndel1-Tara interaction to cell migration, we generated a Tara mutant lacking the domain required for interaction with Ndel1. Tara consists of an N-terminal conserved PH domain (amino acids 6–115) and several coiled-coil regions (amino acids 290–475 and 509–589), the latter of which are responsible for homodimerization. Based on its predicted secondary structure, Tara was divided into three parts (Tara^1–160^, Tara^161–499^, and Tara^500–593^), which were subjected to co-immunoprecipitation with Ndel1. Tara^161–499^ exhibited clear co-immunoprecipitation with Ndel1, while Tara^1–160^ and Tara^500–593^ did not (Fig. 3A, *left*), suggesting that amino acids 161–499 of Tara are important for interaction with Ndel1. Next, we subdivided Tara^161–499^ into four regions (Tara^161–240^, Tara^241–330^, Tara^331–412^, and Tara^413–499^) and analyzed co-immunoprecipitation of the truncated proteins with Ndel1. In this experiment, only Tara^413–499^ showed a considerable interaction with Ndel1 (Fig. 3A, *middle*). Based on these results, two deletion mutants of Tara lacking amino acids 241–330 (Tara^Δ241–330^) or 413–499 (Tara^Δ413–499^) were generated and subjected to co-immunoprecipitation assays with Ndel1. Deletion of amino acids 413–499 of Tara abolished its interaction with Ndel1, while deletion of the 241–330 region did not (Fig. 3A, *right*). These results indicate that amino acids 413–499 of Tara are critical for its interaction with Ndel1.

To determine whether Ndel1-Tara complex formation is essential for regulating cell motility, we performed wound healing and Boyden chamber assays using the Tara^Δ413–499^ construct. In the wound healing assay, the numbers of migrating cells were similar in the presence of wild-type Tara and Tara^Δ413–499^ (Fig. S3B), whereas co-expression of Ndel1 and Tara^Δ413–499^ abolished the migration of cells induced by the intact Ndel1-Tara complex (Fig. 3B,C). No additional increases in migration were observed when Tara^Δ413–499^ was co-expressed with Ndel1 or when the C-terminal tail region of Ndel1 (Ndel1^191–345^) was co-transfected with Tara (Fig. S3C), whereas co-expression of wild-type Ndel1 and Tara enhanced cell motility relative to that of the control (Fig. 3B,C). Since Tara was originally identified as an interaction partner of Trio, which regulates small GTPases including Rac families, we investigated whether the function of Tara was dependent on Rac1 activity. When cells were treated with NSC23766 (50 μM), a Rac1 inhibitor, 12 hours ahead of the scratch, cell migration was mildly decreased in all conditions as expected^46^,^47^, whereas the enhanced migration of Tara-overexpressing cells was maintained (Fig. S3D), implying that Tara’s function in the wound healing is not mainly mediated by Rac1. We also tested the potential influence of Trio on Tara-Ndel1 interaction by performing a series of co-immunoprecipitation experiments in HEK293 cells. When Flag-Trio^1118–1919^ was co-expressed with Myc-Tara and Flag-Ndel1, no significant change in the interaction between Tara and Ndel1 was observed (Fig. S3E). Consistently, there was no prominent change in the co-immunoprecipitation of either wild-type Tara or Tara^Δ413–499^ to Trio (Fig. S3F). Taken together, these results support the notion that a direct interaction between Tara and Ndel1 is critical for regulating cell migration.

### Tara regulates actin remodeling and filopodia formation in association with recruitment of Ndel1 to the peripheral F-actin-rich regions

**Figure 4:**
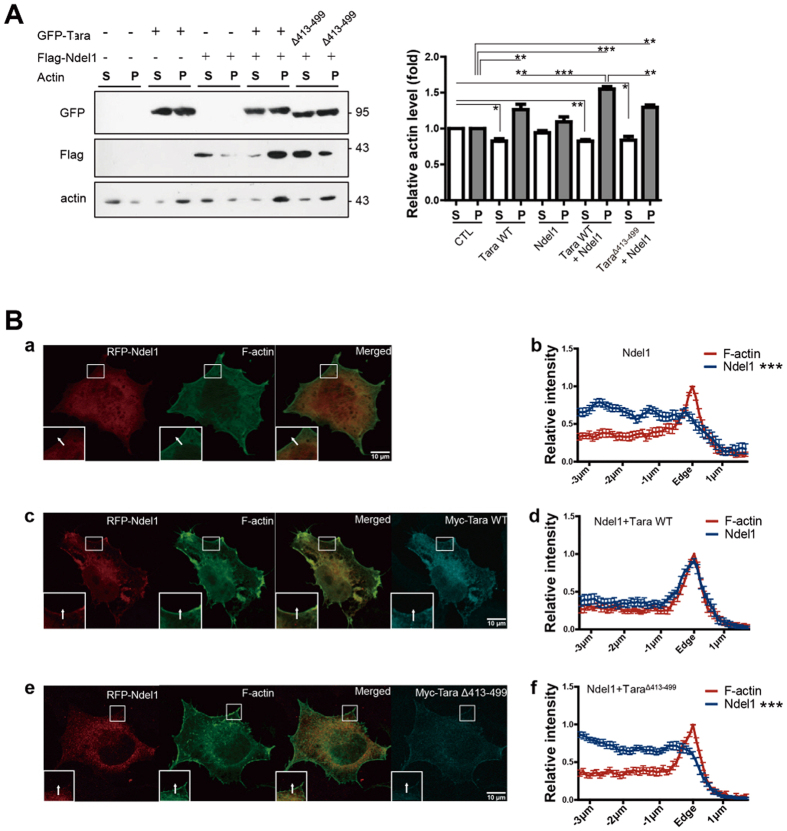
Tara regulates actin dynamics by causing tighter accumulation of Ndel1 into peripheral F-actin rich areas. (**A**) Immunoblot analysis of soluble actin in the supernatant fraction (S) and insoluble actin in the pellet (P) fraction of SH-SY5Y cell lysates transiently expressing Flag-Ndel1 and GFP-Tara. The amounts of G-actin (in the supernatant fraction [S]) and F-actin (in the pellet fraction [P]) were measured by immunoblot analysis using anti-actin antibody. Quantification of the proportions of globular versus pelleted F-actin fractions following ultracentrifugation for the indicated conditions is shown. All results are expressed as mean ± SEM from at least three independent experiments. *p < 0.05, **p < 0.01, ***p < 0.001 by one-way ANOVA with Tukey’s multiple comparison test. (**B**) Co-localization of Ndel1, Tara and F-actin shown by immunofluorescence staining. SH-SY5Y cells expressing RFP-Ndel1 and Myc-tagged constructs of Tara were stained with anti-myc antibody (cyan). (a) Peripheral localizational pattern of RFP-Ndel1 (red) compared to F-actin (green). (b) Quantitative analysis of peripheral co-localizaion of RFP-Ndel1 and F-actin. Intensity profile of three randomly selected fields were measured per each cell (n = 5). (c) Peripheral localizational pattern of RFP-Ndel1 (red) compared to F-actin (green) when Myc-Tara WT was co-expressed. (d) Quantitative analysis of peripheral co-localizaion of Ndel1 and F-actin when Myc-Tara WT was co-expressed. Intensity profile of three randomly selected fields were measured per each cell (n = 5). (e) Peripheral localizational pattern of Ndel1 when Myc-Tara^Δ413–499^ was co-expressed (red) compared to F-actin (green). (f) Quantitative analysis of peripheral co-localizaion of Ndel1 and F-actin when Myc-Tara^Δ413–499^ was co-expressed. Intensity profile of three randomly selected fields were measured per each cell (n = 5). All results are expressed as mean ± SEM from at least three independent experiments. *p < 0.05, **p < 0.01, ***p < 0.001 by two-way ANOVA.

Cell movement is tightly controlled by rapid reorganization of the actin cytoskeleton. Because Tara is an actin-bundling protein that stabilizes F-actin, we examined whether Ndel1 also plays a role in the regulation of actin dynamics via interaction with Tara. When Ndel1 was ectopically expressed in SH-SY5Y cells, no significant change in the level of F-actin was observed, but overexpression of Tara significantly increased the F-actin level when compared to that of the control (Fig. 4A), consistent with previous reports that Tara promotes the stabilization of actin filaments and bundles^35^. Interestingly, co-expression of Ndel1 and Tara additively increased the F-actin level when compared to that of Tara-overexpressed cells (Fig. 4A), suggesting that Ndel1 contributes to actin organization by interacting with Tara. Furthermore, Ndel1 was predominant in the F-actin pellet when co-expressed with wild-type Tara, but not when co-expressed with Tara^Δ413–499^ (Fig. 4A), even though there was no significant difference in the localization pattern between wild-type Tara and Tara^Δ413–499^ (Fig. S4), demonstrating that Ndel1-Tara complex formation is required for the recruitment of Ndel1 to stabilized actin filaments.

Tara-dependent recruitment of Ndel1 to the actin cytoskeleton was further verified by an immunocytochemical analysis of SH-SY5Y cells. Whereas Ndel1 was widely distributed throughout the cells (Fig. 4B, a and b), when co-expressed with wild-type Tara, it was concentrated at the cell boundaries (Fig. 4B, c and d). In addition, Ndel1 co-localized with F-actin and wild-type Tara at the cell periphery region (Fig. 4B, d), consistent with the results of the *in vitro* fractionation assay. This peripheral accumulation of Ndel1 was not induced by co-expression of Tara^Δ413–499^ (Fig. 4B, e and f), indicating that recruitment of Ndel1 to actin filaments was mediated by a direct interaction with Tara. Similarly, peripheral accumulation was only observed with Ndel1^1–190^ and Ndel1^191–345^ showed neither specific co-localization with Tara nor peripheral accumulation (Fig. S4C). Taken together, these results demonstrate that Tara recruits Ndel1 to F-actin structures at the periphery and leading edge of the cell to regulate actin reorganization.

**Figure 5:**
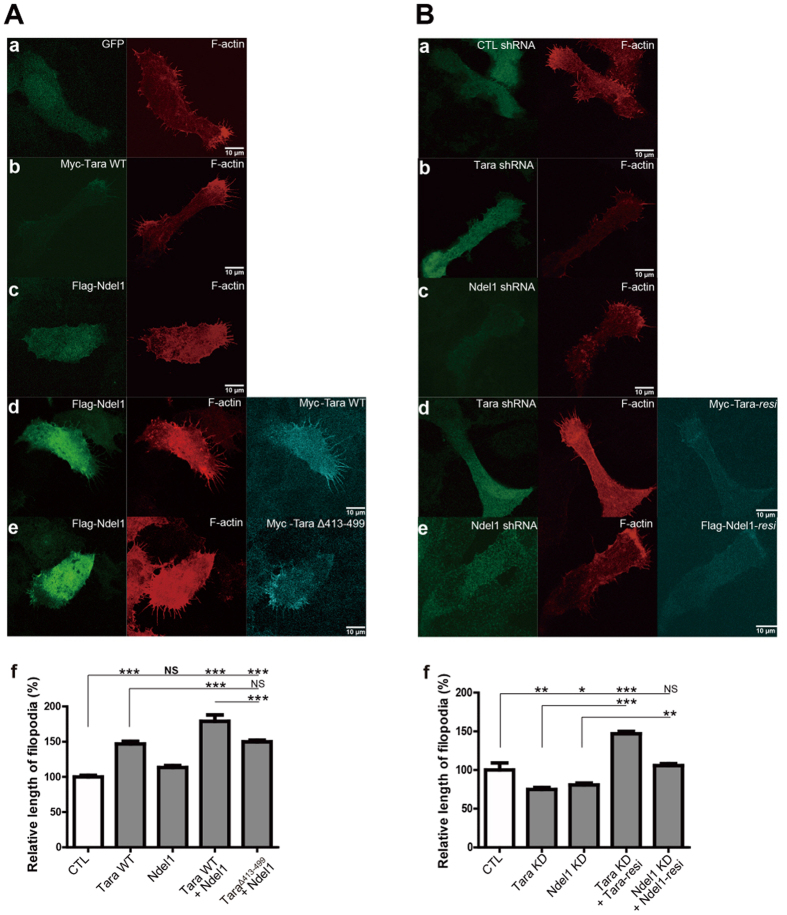
Tara and Ndel1 regulate the length of filopodia in the leading edge of a cell. (**A**) The morphology of filopodia in the leading edge of SH-SY5Y cells expressing Flag-Ndel1 and Myc-Tara visualized by immunofluorescence staining. (a) Cell morphology (green) and F-actin (red) organization in control cells. (b) Effect of Tara expression on filopodia length. Myc-Tara (green) and F-actin (red) are shown. (c) Effect of Ndel1 on filopodia length. Flag-Ndel1 (green) and F-actin (red) are shown. (d) Effect of co-expression of Tara WT and Ndel1 on filopodia length. Flag-Ndel1 (green), F-actin (red), and Myc-Tara WT (cyan) are shown. (e) The effect of co-transfection of Myc-Tara^Δ413–499^ and Flag-Ndel1 on the length of filopodia. Flag-Ndel1 (green), F-actin (red), and Myc-Tara^Δ413–499^ (cyan) are shown. (f) Quantitative analysis of the mean length of filopodia. All results are expressed as mean ± SEM from at least three independent experiments. (**B**) The morphology of filopodia in the leading edge of SH-SY5Y cells upon knockdown of Tara or Ndel1 analyzed by immunofluorescence staining. Transfection of shRNA construct was marked by co-expression of GFP in pLL3.7 vector. (a) Cell morphology (green) and F-actin (red) organization in control cells. (b) The effect of Tara knockdown on the length of filopodia. GFP signal of Tara shRNA (green) and F-actin (red) are shown. (c) The effect of Ndel1 knockdown on the length of filopodia. GFP signal of Ndel1 shRNA (green) and F-actin (red) are shown. (d) Silencing of Tara-induced filopodia decrease was rescued by co-expression of Myc-Tara-*resi,* a RNAi-resistant form of Tara. GFP signal of Tara shRNA (green), F-actin (red), and Myc-Tara-*resi* (cyan) are shown. (e) Silencing of Ndel1-induced filopodia decrease was rescued by co-expression of Flag-Ndel1*-resi*, a RNAi-resistant form of Ndel1. GFP signal of Ndel1 shRNA (green), F-actin (red), and Flag-Ndel1-*resi* (cyan) are shown. (f) Quantitative analysis of the mean length of filopodia. All results are expressed as mean ± SEM from at least three independent experiments. (n = 100 filopodia for each condition) *p < 0.05, **p < 0.01, ***p < 0.001, NS; not significant by one-way ANOVA with Tukey’s multiple comparison test.

To elucidate the effect of the Ndel1-Tara complex on cell migration, we focused on conformational changes of the cytoskeleton such as actin microspikes, filopodia and lamellipodia which push forward the leading edge of a cell to migrate or invade the surrounding tissue. Since formation of these protrusions requires the polymerization and stabilization of actin^1^,^48^,^49^,^50^, we investigated whether the Ndel1-Tara complex is implicated in the process of filopodia formation, elongation or stabilization. Accordingly, filopodia formation in the leading edge of SH-SY5Y cells upon overexpression or knockdown of Tara and Ndel1 was monitored. Tara overexpression led to a dramatic increase in filopodia length relative to that of controls (Fig. 5A, a and b), while expression of Ndel1 showed a relatively mild effect on filopodia elongation (Fig. 5A, c). Interestingly, when wild-type Tara and Ndel1 were co-expressed, average filopodia length was remarkably increased (Fig. 5A, d) whereas co-expression of Ndel1 and Tara^Δ413–499^ abolished the effect (Fig. 5A, e), supporting the notion that Tara and Ndel1 collaborate to regulate filopodia formation and maintenance. The effect on the lifetime of filopodia was less prominent but consistent, while there were no significant changes in the number of filopodia (Fig. S5A, a, b, d, and Movie S1–5). Moreover, filopodia length at the leading edge was significantly decreased when either Ndel1 or Tara was depleted by shRNA-mediated knockdown (Fig. 5B, a–c), an effect which was reversed by co-expression of an RNAi-resistant form of Tara or Ndel1, Tara-*resi* or Ndel1-*resi* (Fig. 5B, d and e). A mild but significant effect on the lifetime of filopodia was also observed (Fig. S5A, a, c, e, and Movie S6–9).

Since Ndel1 has been reported to interact directly and inhibit Cdc42GAP at the leading edge of the cell to stimulate the activity of peripheral Cdc42, a member of Rho family of small GTPases and a central regulator of filopodia dynamics and cell movement^32^,^51^, we investigated the possibility that the Tara and Ndel1 complex influences Cdc42GAP to regulate the activity of Cdc42. When Tara was co-expressed, significant co-localization between Flag-Ndel1 and GFP-Cdc42GAP at the leading edge of SH-SY5Y cell was observed (Fig. S5B), implying their functional association in mediating cell movement. Furthermore, the interaction between Ndel1 and Cdc42GAP was enhanced when Tara WT was co-expressed (Fig. S5C), which was not seen with Tara^Δ413–499^ co-expression. To test whether the interaction between Ndel1 and Tara also influences Cdc42 activity, we applied a PAK1-PBD pull-down assay as a way to assess the active Cdc42 level (Fig. S5D). Interestingly, while overexpression of Tara or Ndel1 only elicited a relatively mild effect on Cdc42 activity, when Tara WT and Ndel1 were co-expressed, the activity of Cdc42 was significantly increased. Furthermore, co-expression of Ndel1 and Tara^Δ413–499^ did not show this phenomenon, supporting the notion that Ndel1 and Tara collaborate to regulate Cdc42 activity. Taken together, these results demonstrate that the Ndel1-Tara complex controls the activity of peripheral Cdc42 and filopodia dynamics at the leading edge of SH-SY5Y cells, likely by reshaping the actin network.

## Discussion

The results presented here indicate that the Ndel1-Tara complex is an important regulator of F-actin organization and filopodia formation during cellular migration (Fig. S6, summary model). Because Ndel1 was initially identified as a component of the Lis1/dynein-containing motor complex, most research on Ndel1 has focused on its microtubule-associated function^16^,^17^. However, there is also evidence suggesting that Ndel1 influences actin filament organization via signaling pathways composed of paxillin, Cdc42GAP and WRC (WAVE regulatory complex)^32^,^34^,^52^. Wu *et al.* showed that Ndel1 is critical for WRC assembly and lamellipodial actin polymerization^52^. Since lamellipodial dynamics is one of the key factors in cell motility, these findings are compatible with our results, thereby leading to a speculation that the Ndel1-Tara complex enhances lamellipodia formation at the leading edge through its function on WRC assembly and stabilizes peripheral filamentous actin and associated structures such as filopodia to stimulate cell movement. Although this sequential actin rearrangement process during cell migration needs to be further investigated, it is plausible that concomitant bindings of WRC (Sra1 and HSPC300) and Tara may exert synergistic effects on cell migration. Moreover, Cdc42GAP and WRC regulate GTPase signaling pathways^32^,^52^, suggesting that Trio, a Rho- and Rac-specific GEF (guanine exchange factor), and Tara may cooperate in the small GTPase signaling pathways. Trio activates Rho/Rac GTPases by promoting the exchange of GDP for GTP to enhance cell adhesion and movement likely through reorganization of the actin cytoskeleton^51^,^53^. Thus, these findings provide a basis for speculation that Tara-Ndel1 may establish a functional link between Trio and its particular effects on Rho- and Rac-GTPases within the cell periphery, justifying further investigation of detailed mechanisms linking the Ndel1-Tara complex to GTPase signaling pathways. Taken together, our results provide additional mechanistic insight supporting the view that Ndel1 controls cellular movement by regulating actin filament dynamics.

Our data also demonstrated that the interaction between Ndel1 and Tara is mediated by the N-terminal coiled-coil region of Ndel1 (amino acids 1–190). Notably, this N-terminal region is also important for the binding of Ndel1 to Lis1 54. Thus, it is tempting to speculate that Tara may not only recruit Ndel1 to the actin-rich cell periphery, but may also regulate the binding of Ndel1 to the Lis1/dynein complex, thereby providing leverage to promote the actin-related function of Ndel1. It will also be interesting to determine whether these two processes are interlinked to elucidate the mechanism involved.

Ndel1 has been implicated in the neurodevelopmental aspects of mental illness such as schizophrenia^55^,^56^; this role is thought to be accomplished through its interaction with DISC1 16,17,18,25. In this regard, the discovery of Tara as a novel Ndel1-interacting protein further characterizes the roles of actin-bundling proteins in neuronal development and related pathological conditions. Indeed, a recent study suggested that Tara is a potential susceptibility factor for schizophrenia that has the capability to alter the morphology of neuron-like cells^57^. It has also been widely hypothesized that the disturbances in auditory processing occur frequently in schizophrenic patients compared with normal subjects^58^,^59^. Although controversy exists in the association between sensory inputs and schizophrenia, there are a number of clinical and genetic studies reporting a contribution of hearing impairment to psychiatric disorders^59^,^60^,^61^, implying the significant influence of auditory stimuli in neurodevelopmental processes. Given that mutations in the *Tara* locus are associated with deafness^42^,^43^, it is noteworthy that there is a high comorbidity between hearing defects and psychiatric disorders such as schizophrenia in the general population^58^,^59^,^60^,^61^. Thus understanding the direct interaction between Ndel1 and Tara may provide clues to the molecular mechanisms underlying this association.

## Materials and Methods

### Plasmids

For yeast two-hybrid assays, a human nuclear distribution gene E homolog (A. nidulans)-like 1 (Ndel1) cDNA was amplified by PCR followed by subcloning into pPC86 vector to make pPC86-Ndel1. To construct the disrupted-in-schizophrenia 1 (DISC1), Trio^1118–1919^, and Tara plasmids, a region of human DISC1, Trio^1118–1919^, and Tara corresponding to the designated codons were amplified by PCR and inserted into the Sal I and Not I sites of the pPC97 vector. For constructs of full length human Ndel1 and fragments, corresponding regions of human Ndel1 (hNdel1) were amplified from reverse transcription products generated from HEK293 cells and inserted into the pEGFP-C3 (Clontech), and pFlag-CMV (Sigma). For human Tara constructs, the full-length Tara coding sequence was amplified using reverse transcription products generated from HEK293 cells, subcloned into pEGFP-C3 and pCDNA3.1 Myc-His (Invitrogen). For human Cdc42GAP and Lis1 construct, the full-length Cdc42GAP and Lis1 coding sequences were amplified from reverse transcripts of HEK293 cell mRNA and subcloned into pEGFP-C3 (Clontech). To construct the deletion mutant of Tara, a region of human Tara corresponding to the designated codon was amplified by PCR using pEGFP-Tara plasmid as template and inserted into the pEGFP-C3. Sequences were verified by DNA sequencing. For shRNA constructs, human Ndel1 shRNA was prepared following previous description^24^. The oligonucleotide sequences used for the constructs are GCAGGTCTCAGTGTTAGAA for human Ndel1 and GCTGACAGATTCAAGTCTCAA for human Tara. These oligonucleotides were annealed and ligated into the pLentiLox3.7 vector using Hpa I and Xho I sites.

### Yeast Two-Hybrid Screening and interaction analysis

A human Ndel1 cDNA was amplified by PCR and then cloned into pPC97 vector to make pPC97-Ndel1. MaV203 yeast cells were co-transformed with pPC97-Ndel1 and human fetal brain cDNA library plasmids cloned in pPC86 (GibcoBRL). A total of 3 × 10^6^ co-transformants was initially screened for growth on *Leu*^*−*^*, Trp*^*−*^, and *His*^*−*^ media containing 20 mM of 3-amino-1,2,4-triazol (3-AT; Sigma). The plasmids were isolated from the positives, amplified in *DH5α*, and analyzed by DNA sequencing. For constructs for Tara, designated regions of human Tara was amplified by PCR and inserted into the Sal I and Not I sites of the pPC86 vector. MaV203 yeast cells were co-transformed with various constructs into pPC97 and pPC86 as indicated. Colonies grown on synthetic defined media (SD)-*Leu*^−^*/Trp*^*−*^ Plates were streaked onto yeast peptone dextrose (YPD) plates, and colony-lifting assays for β-galactosidase expression were carried out. For growth test on the selective media, transformants resuspended in distilled water were dropped onto a dried SD-*Leu*^−^/*Trp*^−^ plate containing 20 mM 3-AT and incubated for 3 days at 30 °C.

### Cell culture and transfection

Cells were grown in DMEM or MEM supplemented with 10% fetal bovine serum and antibiotics under 5% CO_2_ at 37 °C. Cells were transfected using Lifopectamine 2000 (Invitrogen) or Vivamagic (VIVAGEN) according to the manufacturer’s instruction.

### Antibodies and immunoblot analysis

A rabbit polyclonal anti-GFP protein (GFP; Molecular Probes), anti-Flag (ABR) and mouse monoclonal anti-flag M2 (SIGMA) antibodies were used. Anti-actin (I-19), anti-GAPDH (6C5), anti-GFP (B-2) and anti-Myc (9E10) antibodies were purchased from Santa Cruz Biotechnology. Anti-Tara antibody and Anti-Ndel1 antibody were purchased from Thermo scientific (PA5-29092) and Proteintech (17262-1-AP). Anti-Trio antibody and Anti-DISC1 antibody were purchased from Santa Cruz Biotechnologies (D-20) and Millipore (Q2269477), respectively. For immunoblotting, cells were lysed in Nonidet P-40 (NP-40) lysis buffer (50 mM Tris, pH 8.0; 150 mM NaCl, 1% NP-40, 5 mM EDTA, 5 mM glycerol-2-phosphate, 2 mM sodium pyrophosphate, 5 mM NaF, 2 mM Na_3_VO_4_, 1 mM DTT, EDTA-free protease inhibitor mixture [Roche]) and pre-cleared by centrifugation for 10 min at 12,000 g. Supernatants were denatured in SDS sample buffer by boiling for 5 min and subjected to sodium dodecyl sulfate polyacrylamide gel electrophoresis (SDS-PAGE), followed by immunoblotting. For detection, films or the LAS 4000 (Fuji film, Tokyo, Japan) were used. For quantification, images were acquired with a Luminescent image analyzer Las-4000 (Fujifilm) and analyzed with image J software.

### Immunoprecipitation

Cultured cells were homogenized in NP-40 lysis buffer (50 mM Tris, pH8.0, 150 mM NaCl, 1% NP-40, 5 mM EDTA, 5 mM NaF, 2 mM Na_3_VO_4_, 1 mM DTT, EDTA-free protease inhibitor mixture [Roche]) and precleared by centrifugation for 15 min at 12,000 g. The supernatant was incubated with 0.5–1 μg of antibodies on a rocking platform for 3 hours at 4 °C; 100 μL of 10% protein-A agarose (GE Healthcare) resuspended in the same lysis buffer were added and incubated for an additional 90 min at 4 °C with gentle shaking. The precipitate was washed three times with lysis buffer and resuspended in the 2x SDS sample loading buffer.

### Immunocytochemistry

SH-SY5Y cells cultured on coverslips were washed one time in PBS and fixed by immersion in cold 4% paraformaldehyde/PBS for 30 min. For detection of Tara and Ndel1, the fixed cells were incubated for 30 min in the blocking solution (2% horse serum, 1% Triton X-100 in PBS). The cells were then rinsed three times with PBS at room temperature. After permeabilization, cells were incubated with the mouse anti-myc (1:200, Santa cruz) and rabbit anti-flag (1:1000, ABR) antibodies for 6 hours, and the secondary antibodies, Alexafluor 488-conjugated goat anti-rabbit IgG and Alexafluor 568-conjugated goat mouse IgG (1:500; Molecular Probes), were incubated for 1 hour at room temperature in the blocking solution. The spatial patterns of F-actin were analyzed with co-expression of the actin-binding calponin homology domain of utrophin (UtrCH) fused to GFP or RFP. The cells on coverslips were rinsed three times with PBS and mounted in the Antifade medium (Molecular Probes). The pictures were taken with confocal microscope (Olympus, FluoView-1000).

For the sequential immunostaining of endogenous Tara and Ndel1, cells were incubated with rabbit anti-Tara antibody (1:500, Thermo scientific) for 2 hours at room temperature followed by two rounds of incubation with Alexafluor 568-conjugated goat anti-rabbit secondary antibody for 1 hour each round at room temperature^62^. After first staining, cells were incubated with rabbit anti-Ndel1 (1:500) antibody for overnight at 4 °C followed by incubation with Alexafluor 488-conjugated goat anti-rabbit secondary antibody for 30 minutes at room temperature. For the control staining, the same procedure was taken without anti-Ndel1 antibody incubation.

For quantification of filopodial length, filopodia were defined as thin (<1 μm wide) UtrCH-positive protrusions that extended at least 0.75 μm from the leading edge of SH-SY5Y cells^63^. At least 10 microscope fields were captured for each transfection condition and the images were analyzed by Image J software (http://imagej.nih.gov/ij/).

### *In vitro* scratch wound healing assay

Cell migration was measured by *in vitro* scratch assay. 5 × 10^5^ cells were plated in a 12-well plate one day before transfection. When cells reached 95~100% of confluency in MEM with 10% FBS, the medium was replaced with fresh serum-free MEM to inhibit further proliferation. At least 24 hours after transfection (72 hours after transfection for knockdown conditions), the cell monolayer was scraped in a straight line to create a “scratch” with a p1000 pipet tip. The number of migrated cells was counted at 10 hours after scratch using image J software. When applicable, SH-SY5Y cells are treated with actinomycin-D (1 μM, Sigma Aldrich) for 24 hours ahead of scratch or NSC23766 (Rac1 inhibitor, 50 μM, Sigma Aldrich) for 12 hours ahead of scratch.

### Boyden chamber assay

SH-SY5Y cells (2 × 10^4^ cells/50 μL medium) were placed in the upper chamber of Boyden chamber with serum-free medium. The lower chamber contained 35 μL of medium supplemented with 10% FBS. After 24 hours of incubation (72 hours after transfection for knockdown conditions), the migrated cells on the lower surface of the membranes were fixed with chilled methanol and stained with crystal violet. The migrated cells were counted in at least three to four randomly selected microscopic fields on the membrane and the results are summarized and expressed as the mean number of migrated cells ± SEM per microscopic field.

### Actin fractionation analysis

Transfected cells were lysed with buffer containing 10 mM Tris, pH 7.4; 2 mM MgCl2, 1% Triton X-100, 0.2 mM DTT and 15% glycerol. Soluble (G-actin) and insoluble (F-actin) fractions were separated by centrifugation (100,000 g, 1 hour) at 4 °C. Each fraction was resolved by 10% SDS-PAGE and subjected to immunoblot analysis with actin antibody (1:500, Santa Cruz).

### qRT-PCR analysis

Total RNA was isolate form cultured SH-SY5Y cells using TRI Reagent (Ambion). cDNA synthesis was conducted with the High-Capacity RNA-to-cDNA Kit and qPCR conducted with Power SYBR Green Master Mix (Life Technologies) using the StepOnePlus thermocycler (Applied Biosystems). Primers used for qRT-PCR are as the following; 5′-TGCAGCAGGAGAAGGAGTGG-3′ (forward) and 5′-GTTGTGCAGCAGCTC-3 (reverse) for Tara; 5′-TCCCGAACTGCTAGTTCTTGCC-3′ (forward) and 5′-GGCTGAACAAAGAAATAGAGA-3′ (reverse) for Ndel1. Results shown are the mean of at least three independent experimental replicates.

### Blot Overlay assays

GST fusion proteins (GST-Tara WT and GST-Tara^Δ413–499^) and His-Ndel1 were expressed in BL21 bacteria and purified with glutathione-Sepharose beads. For the blot overlay assay, 2 μg of GST or GST-Tara fragments were separated by SDS/PAGE and then transferred to a PVDF membrane. The membrane was blocked in 10% skim milk in Tris buffered saline (TBS)-Tween overnight at 4 °C and was further incubated with 1 μg/mL of His-Ndel1 in a blot overlay buffer (TBS-Tween, 0.1% BSA, and 2 mM MgSO_4_) for 3 hours. The membrane-bound proteins were cross-linked with 0.2% glutaldehyde for 15 min and then washed extensively, and they were subjected to immunoblot analyses.

### Time-lapse imaging analysis and quantification

For quantification of filopodia lifetime, a live time-lapse imaging was carried out using spinning disc confocal microscope (Olympus, IX81). SH-SY5Y cells were imaged for 20 min with 10 sec intervals. Filopodia lifetime was determined by following individual filopodia from appearance from the cell membrane to disappearance. All images were collected and processed using MetaMorph software. (Molecular Devices).

### Assays for Cdc42 Activity

Cdc42 activity was monitored by measuring the GTP-bound Cdc42 precipitated using PAK1-PBD agarose (Cell biolabs). In brief, SH-SY5Y cells which transiently express Ndel1 or Tara constructs were harvested and homogenized by sonication. After centrifugation, lysates were incubated with PAK1-PBD beads for 1 hour at 4 °C and after incubation in a rotary stage, proteins bound on beads were subjected to immunoblot analysis with anti-Cdc42 antibody (Cell biolabs).

### Statistical analysis

Data were analyzed using the GraphPad Prism 5 software (http://www.graphpad.com/ scientific-software/prism/) and presented as the mean ± SEM. Statistical significance was determined by ANOVA or student’s t-test.

## Additional Information

**How to cite this article**: Hong, J.-H. *et al.* Regulation of the actin cytoskeleton by the Ndel1-Tara complex is critical for cell migration. *Sci. Rep.* **6**, 31827; doi: 10.1038/srep31827 (2016).

## Supplementary Material

Supplementary Information

Supplementary Movie S1

Supplementary Movie S2

Supplementary Movie S3

Supplementary Movie S4

Supplementary Movie S5

Supplementary Movie S6

Supplementary Movie S7

Supplementary Movie S8

Supplementary Movie S9
