# Supplementary material for: Regulation of the actin cytoskeleton by the Ndel1-Tara complex is critical for cell migration
Source: Sci Rep. 2016 Aug 22;6:31827. doi: 10.1038/srep31827 (PMC4992831; doi:10.1038/srep31827)
Supplement: Supplementary Information [file srep31827-s1.pdf]

Regulation of the actin cytoskeleton by the Ndel1-Tara complex is critical for cell migration

Ji-Ho Hong[1a], Yongdo Kwak[1a+], Youngsik Woo[1], Cana Park[1], Seol-Ae Lee[1], Haeryun Lee[1], Sung Jin Park[1], Yeongjun Suh[1], Bo Kyoung Suh[1], Bon Seong Goo[1], Dong Jin Mun[1], Kamon Sanada[2], Minh Dang Nguyen[3], and Sang Ki Park[1]*

From the [1]Department of Life Sciences, Pohang University of Science and Technology, Pohang 790-784, Republic of Korea, the [2]Molecular Genetics Research Laboratory, University of Tokyo, 7-3-1 Hongo, Bunkyo-ku, Tokyo 113-0033, Japan, and the [3]Hotchkiss Brain Institute, Departments of Clinical Neurosciences, Cell Biology and Anatomy, and Biochemistry and Molecular Biology, University of Calgary, Calgary T2N 4N1, Canada

[a)] These authors contributed equally to this work.

*) Correspondences should be addressed to SKP at skpark@postech.ac.kr.

+) Current address: Psychiatry research team. SK biopharmaceutics Ltd., Republic of Korea

Figure S1.

(A) Interaction of Tara and Trio in a yeast two-hybrid assay.

(B) Co-IP of Flag-Ndel1 with Myc-Tara. Anti-Flag immunoprecipitates were analyzed by immunoblotting with anti-Flag and anti-Myc.

(C) Co-IP of endogenous Tara and Ndel1 from SH-SY5Y cell lysates. Anti-Ndel1 immunoprecipitates were analyzed by immunoblotting with anti-Tara and anti-Ndel1.

(D) Co-IP of endogenous Tara and Ndel1 from SH-SY5Y (a) and HEK293 cell lysates (b). Anti-Tara immunoprecipitates were analyzed by immunoblotting with anti-Tara, anti-Ndel1, and anti-Trio antibodies as indicated.

(E) Co-localization of Tara and Ndel1 at the leading edge of SH-SY5Y cells (white arrow). Endogenous Tara and Ndel1 were stained with anti-Tara (red) and anti-Ndel1 antibodies (green).

(F) To prevent the possibility of cross-reaction between endogenous Tara and Ndel1 staining sequences in (E), incubation step with Ndel1 antibody was skipped after Tara staining (Alexa 568, red signal). When the first Ndel1 antibody was omitted, no fluorescence was observed with Alexa 488 (green signal).

(G) To test the specificity of antibodies to Tara and Ndel1, immunocytochemistry was performed with Myc-Tara- or Flag-Ndel1-transfected SH-SY5Y cells. (a) Myc-Tara was detected with anti-Tara (red) and anti-Myc (green) (Pearson's coefficient 0.9563 ± 0.009). (b) Flag-Ndel1 was detected with anti-Ndel1 (red) and anti-Flag (green) (Pearson's coefficient 0.9613 ± 0.006).

(H) Immunocytochemistry to confirm co-localization between Tara/Trio and Ndel1/DISC1. (a) Endogenous Tara and Trio were stained with anti-Tara (red) anti-Trio antibodies (green). (b) Endogenous Ndel1 and DISC1 were stained with anti-Ndel1 (red) and anti-DISC1 antibodies

(green).

(I) Co-IP of Flag-Ndel1, Myc-Tara and GFP-DISC1. Anti-Flag immunoprecipitates were analyzed by immunoblotting with anti-Flag, anti-Myc and anti-GFP.

(J) Co-IP of Flag-Ndel1, Myc-Tara and GFP-Lis1. Anti-Flag immunoprecipitates were analyzed by immunoblotting with anti-Flag, anti-Myc and anti-GFP.

(K) Co-IP of Myc-Tara, Flag-Trio$^{1118-1919}$, and Flag-Ndel1. Anti-Myc immunoprecipitates were analyzed by immunoblotting with anti-Flag, anti-Myc.

(L) Interaction of Ndel1 with Tara WT *in vitro* analyzed by a blot overlay assay. The PVDF membrane with the purified wild-type GST-Tara WT and His-Ndel1 protein bands transferred from an SDS/PAGE was first stained with Coomassie Blue R250 *(Left)* and subjected to GST-Tara blot overlay followed by anti-His immunoblot analyses *(Right)*.

Figure S2.

(A) Characterization of Tara shRNA construct. The target sequences for shRNA constructs were control, 5'-AAACTACCGTTGTATAGGTGT; Tara 5'- GCTGACAGATTCA -AGTCTCAA. (a) Endogenous Tara mRNA level in Tara knockdown SH-SY5Y cells. SH-SY5Y cells were transfected with shRNA constructs and incubated for 72 hours. hTara mRNA levels were measured by qRT-PCR and normalized to GAPDH mRNA. (b) Knockdown of endogenous Tara by Tara shRNA in SH-SY5Y cells. SH-SY5Y cells were transfected with control or hTara shRNA constructs and incubated for 72 hours. Endogenous Tara protein levels were analyzed by anti-Tara immunoblotting. All results are expressed as mean ± SEM from at least three independent experiments. *; $p < 0.05$, **; $p < 0.01$, ***; $p < 0.001$ by student's t-test.

(B) Characterization of Ndel1 shRNA construct. The target sequence for Ndel1 shRNA was

5’- GCAGGTCTCAGTGTTAGAA. (a) Endogenous Ndel1 mRNA level in Ndel1 knockdown SH-SY5Y cells. hNdel1 mRNA levels were measured by qRT-PCR and normalized to GAPDH mRNA. (b) Knockdown of endogenous Ndel1 by Ndel1 shRNA. SH-SY5Y cells were transfected with control or hNdel1 shRNA constructs and incubated for 72 hours. Endogenous Ndel1 protein levels were analyzed by anti-Ndel1 immunoblotting. All results are expressed as mean ± SEM from at least three independent experiments. *; $p < 0.05$, **; $p < 0.01$, ***; $p < 0.001$ by student’s t-test.

(C) Expression of shRNA-resistant Tara construct. HEK293 cells were transfected with Flag-hTara or Flag-hTara-*resi* constructs in combination with hTara shRNA constructs were co-transfected. *; $p < 0.05$, **; $p < 0.01$, ***; $p < 0.001$ by student’s t-test.

(D) Expression of shRNA-resistant Ndel1. HEK293 cells were transfected with Flag-hNdel1 or Flag-hNdel1-*resi* constructs in combination with hNdel1 shRNA constructs were co-transfected. *; $p < 0.05$, **; $p < 0.01$, ***; $p < 0.001$ by student’s t-test.

(E and F) The effect of (E) Tara overexpression on Ndel1-deficient SH-SY5Y cells and (F) Ndel1 overexpression on Tara-deficient SH-SY5Y cells in the wound healing assay. All results are expressed as mean ± SEM from at least three independent experiments. *; $p < 0.05$, **; $p < 0.01$, ***; $p < 0.001$ by one-way ANOVA with Tukey’s multiple comparison test.

Figure S3.

(A) The effect of actinomycin D on wound healing of SH-SY5Y cells expressing Tara and Ndel1. Dashed white lines indicate the initial boundaries of the scratches (0 hour) and yellow lines indicate front lines of moving cells at indicated time points. Over 200 cells were counted for each cell group. All results are expressed as mean ± SEM from at least three independent experiments. *; $p < 0.05$, **; $p < 0.01$, ***; $p < 0.001$, NS; not significant by one-

way ANOVA with Tukey's multiple comparison test.

(B) The effect of the deletion mutants of Tara on wound healing of SH-SY5Y cells. Cells transiently expressing GFP-Tara or GFP tagged deletion mutants of Tara were tanked at 0 and 10 hours during scratch wound-healing assay. All results are expressed as mean ± SEM from at least three independent experiments. *; $p < 0.05$, **; $p < 0.01$, ***; $p < 0.001$, NS; not significant by one-way ANOVA with Tukey's multiple comparison test.

(C) The effect of C-terminal tail region of Ndel1 (amino acids 191–345; Ndel1$^{191\text{-}345}$) expression in wound healing assay. All results are expressed as mean ± SEM from at least three independent experiments. *; $p < 0.05$, **; $p < 0.01$, ***; $p < 0.001$, NS; not significant by one-way ANOVA with Tukey's multiple comparison test.

(D) The effect of NSC23766 (used as Rac1 inhibitor) in wound healing assay. Representative images of cells tanked at 0 and 10 hours during scratch wound-healing assay. Over 200 cells were counted for each cell group. All results are expressed as mean ± SEM from at least three independent experiments. *; $p < 0.05$, **; $p < 0.01$, ***; $p < 0.001$, NS; not significant by one-way ANOVA with Tukey's multiple comparison test.

(E) Co-immunoprecipitation of Myc-Tara, Flag-Ndel1, and Flag-Trio$^{1118\text{-}1919}$. Anti-Myc immunoprecipitates were analyzed by immunoblotting with anti-Flag, anti-Myc.

(F) Co-immunoprecipitation of Flag-Trio$^{1118\text{-}1919}$ with Myc-Tara WT or Myc-Tara$^{\Delta 413\text{-}499}$. Anti-Myc immunoprecipitates were analyzed by immunoblotting with anti-Flag, anti-Myc.

Figure S4.

(A) Actin fractionation analysis of SH-SY5Y cell lysates upon expression of GFP-Tara constructs. The lysates were centrifuged at 100,000g for 1 hour, and the pellets were used to measure the F-actin. The amounts of G-actin (in the supernatant fraction [S]) and F-actin (in

the pellet fraction [P]) were detected by immunoblot analysis using anti-actin antibody. The expression of Tara in the pellet fractions following ultracentrifugation were quantified. All results are expressed as mean ± SEM from at least three independent experiments. *; $p < 0.05$, **; $p < 0.01$, ***; $p < .001$, NS; not significant by student's t-test.

(B) Co-localization Tara and F-actin shown by immunofluorescence staining. (a) The morphology of SH-SY5Y cell expressing Myc-Tara WT. Myc-Tara WT (green) and F-actin (red) are shown. (b) Quantitative analysis of peripheral co-localization of Myc-Tara WT and F-actin. Intensity profile of three randomly selected fields were measured per each cell (n=5). (c) The morphology of SH-SY5Y cell expressing Myc-Tara$^{\Delta 413\text{-}499}$. Myc-Tara$^{\Delta 413\text{-}499}$ (green) and F-actin (red) are shown. (d) Quantitative analysis of peripheral co-localization of Myc-Tara$^{\Delta 413\text{-}499}$ and F-actin. Intensity profile of three randomly selected fields were measured per each cell (n=5). All results are expressed as mean ± SEM from at least three independent experiments.

(C) Co-localization Ndel1 and F-actin shown by immunofluorescence staining. (a) The morphology of SH-SY5Y cell expressing GFP-Ndel1$^{1\text{-}190}$ and Myc-Tara. GFP-Ndel1$^{1\text{-}190}$ (green) and F-actin (red) are shown. (b) Quantitative analysis of peripheral co-localization of GFP-Ndel1$^{1\text{-}190}$ and F-actin. Intensity profile of three randomly selected fields were measured per each cell (n=5). (c) The morphology of SH-SY5Y cell expressing GFP-Ndel1$^{191\text{-}345}$ and Myc-Tara. GFP-Ndel1$^{191\text{-}345}$ (green) and F-actin (red) are shown. (d) Quantitative analysis of peripheral co-localization of GFP-Ndel1$^{191\text{-}345}$ and F-actin. Intensity profile of three randomly selected fields were measured per each cell (n=5). All results are expressed as mean ± SEM from at least three independent experiments. ***; $p < .001$ by two-way ANOVA.

Figure S5.

(A) Live images to visualize filopodia dynamics in SH-SY5Y cells. Cells were transfected as

indicated and cultured in ibidi glass-bottomed 35mm dishes for 24 hours before live-cell imaging. (a) Montages of single frames from 420 sec of live-cell microscopy time series. Arrows indicate filopodia. (b and c) Quantification of lifetime of filopodia from experiments shown in (a). (d and e) Quantitative analysis of the average number of filopodia. All results are expressed as mean ± SEM from at least three independent experiments. (n=80 filopodia for each condition) *; $p < 0.05$, **; $p < 0.01$, ***; $p < 0.001$, NS; not significant by one-way ANOVA with Tukey's multiple comparison test.

(B) Co-localization of Cdc42GAP, Ndel1, and Tara at the leading edge (arrows) of SH-SY5Y cells. Myc-Tara was stained with anti-Myc antibody. GFP-Cdc42GAP (green), RFP-Ndel1 (red), and Myc-Tara (cyan) were shown.

(C) Interaction of Ndel1 and Cdc42GAP upon Tara co-expression. Anti-Flag immunoprecipitates were analyzed by immunoblotting with anti-GFP and anti-Flag antibodies. Band intensities of GFP-Cdc42GAP in the immunoprecipitates were analyzed. All results are expressed as mean ± SEM from at least three independent experiments. ***; $p < 0.001$ by one-way ANOVA with Tukey's multiple comparison test.

(D) Quantitative analysis of Cdc42 activity. SH-SY5Y cells expressing Ndel1 or Tara transiently were subjected to immunoblot analysis with anti-Cdc42 antibody (Cell biolabs, Catalog #240201). Activated form of Cdc42 were subjected to PAK1-PBD agarose (GTP-CDC42) for 1 hour at 4°C. All results are expressed as mean ± SEM from at least three independent experiments. *; $p < 0.05$, **; $p < 0.01$, ***; $p < 0.001$ by one-way ANOVA with Tukey's multiple comparison test.

Figure S6.

A schematic model describing a role for Ndel1-Tara complex in the process of cell migration through regulation of cellular actin dynamics, the activity of peripheral GTPases, and

filopodia formation.

Movie S1.

F-actin dynamics in control SH-SY5Y cells. Movie represents total time of 20 minutes. RFP-UtrCH constructs were used to display peripheral F-actin dynamics during filopodia formation. Corresponds to image in figure S5A.

Movie S2.

F-actin dynamics in Tara-overexpressed SH-SY5Y cells. Movie represents total time of 20 minutes. RFP-UtrCH constructs were used to display peripheral F-actin dynamics during filopodia formation. Corresponds to image in figure S5A.

Movie S3.

F-actin dynamics in Ndel1-overexpressed SH-SY5Y cells. Movie represents total time of 20 minutes. RFP-UtrCH constructs were used to display peripheral F-actin dynamics during filopodia formation. Corresponds to image in figure S5A.

Movie S4.

F-actin dynamics in wild-type Tara and Ndel1-overexpressed SH-SY5Y cells. Movie represents total time of 20 minutes. RFP-UtrCH constructs were used to display peripheral F-actin dynamics during filopodia formation. Corresponds to image in figure S5A.

Movie S5.

F-actin dynamics in Tara$^{\Delta 413\text{-}499}$ and Ndel1-overexpressed SH-SY5Y cells. Movie represents total time of 20 minutes. RFP-UtrCH constructs were used to display peripheral F-actin

dynamics during filopodia formation. Corresponds to image in figure S5A.

Movie S6.

F-actin dynamics in Tara-knockdown SH-SY5Y cells. Movie represents total time of 20 minutes. RFP-UtrCH constructs were used to display peripheral F-actin dynamics during filopodia formation. Corresponds to image in figure S5A.

Movie S7.

F-actin dynamics in Ndel1-knockdown SH-SY5Y cells. Movie represents total time of 20 minutes. RFP-UtrCH constructs were used to display peripheral F-actin dynamics during filopodia formation. Corresponds to image in figure S5A.

Movie S8.

F-actin dynamics in Tara-knockdown SH-SY5Y cells co-transfected with Tara-*resi* constructs for rescue experiments. Movie represents total time of 20 minutes. RFP-UtrCH constructs were used to display peripheral F-actin dynamics during filopodia formation. Corresponds to image in figure S5A.

Movie S9.

F-actin dynamics in Ndel1-knockdown SH-SY5Y cells co-transfected with Ndel1-*resi* constructs for rescue experiments. Movie represents total time of 20 minutes. RFP-UtrCH constructs were used to display peripheral F-actin dynamics during filopodia formation. Corresponds to image in figure S5A.

FIGURE S1

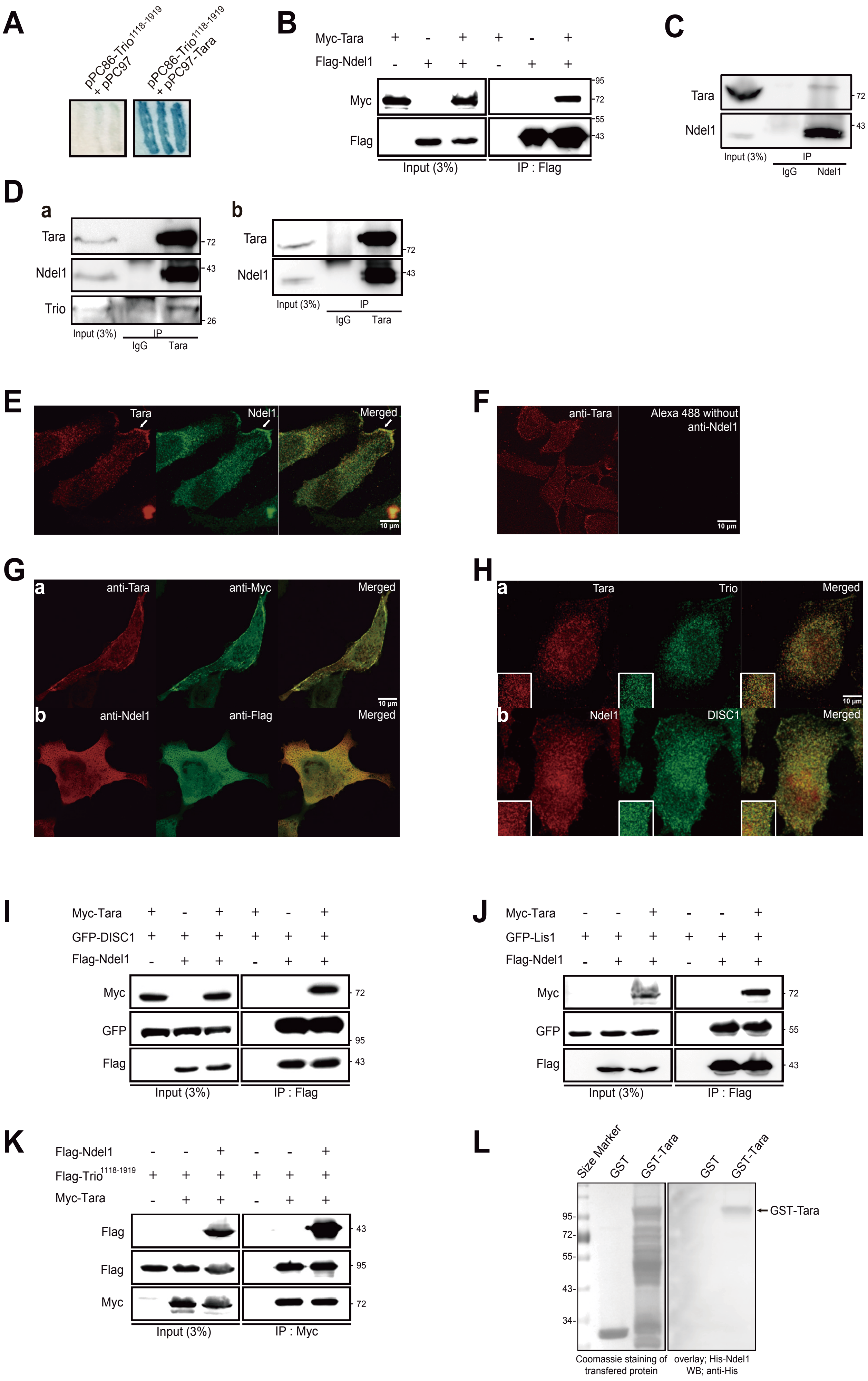
A
pPC86-Trio1118-1919 + pPC97
pPC86-Trio1118-1919 + pPC97-Tara
B
Myc-Tara
Flag-Ndel1
Myc
Flag
Input (3%)
IP : Flag
C
Tara
Ndel1
Input (3%)
IP
IgG
Ndel1
D
a
Tara
Ndel1
Trio
Input (3%)
IP
IgG
Tara
b
Tara
Ndel1
Input (3%)
IP
IgG
Tara
E
Tara
Ndel1
Merged
10 μm
F
anti-Tara
Alexa 488 without anti-Ndel1
10 μm
G
a
anti-Tara
anti-Myc
Merged
10 μm
b
anti-Ndel1
anti-Flag
Merged
H
a
Tara
Trio
Merged
10 μm
b
Ndel1
DISC1
Merged
I
Myc-Tara
GFP-DISC1
Flag-Ndel1
Myc
GFP
Flag
Input (3%)
IP : Flag
J
Myc-Tara
GFP-Lis1
Flag-Ndel1
Myc
GFP
Flag
Input (3%)
IP : Flag
K
Flag-Ndel1
Flag-Trio1118-1919
Myc-Tara
Flag
Flag
Myc
Input (3%)
IP : Myc
L
Size Marker
GST
GST-Tara
GST
GST-Tara
GST-Tara
Coomassie staining of transfered protein
overlay; His-Ndel1 WB; anti-His

FIGURE S2

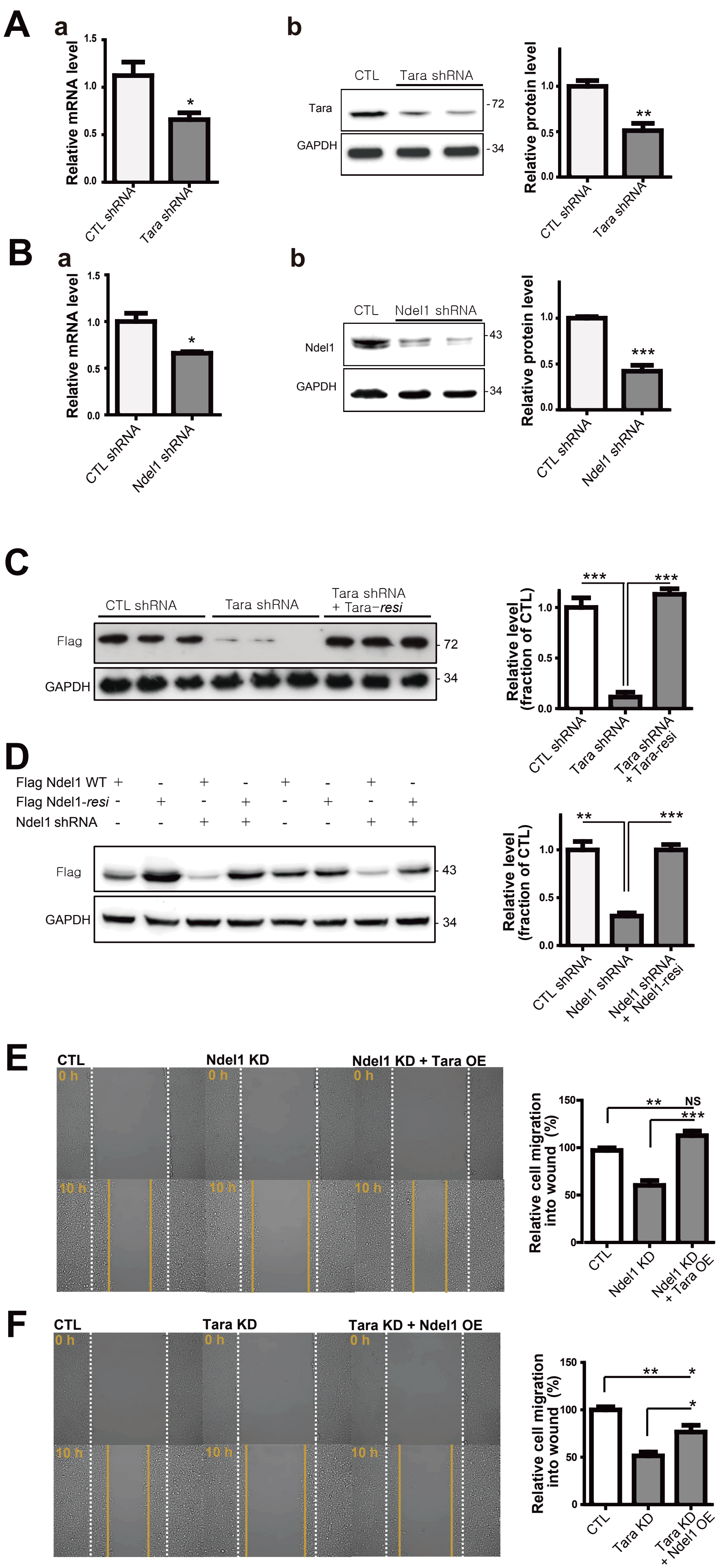
A
a
Relative mRNA level
CTL shRNA
Tara shRNA
b
CTL
Tara shRNA
Tara
GAPDH
72
34
Relative protein level
B
Ndel1 shRNA
Ndel1
43
C
CTL shRNA
Tara shRNA + Tara-resi
Flag
Relative level (fraction of CTL)
D
Flag Ndel1 WT
Flag Ndel1-resi
Ndel1 shRNA + Ndel1-resi
E
CTL
Ndel1 KD
Ndel1 KD + Tara OE
0 h
10 h
Relative cell migration into wound (%)
NS
F
Tara KD
Tara KD + Ndel1 OE

FIGURE S3

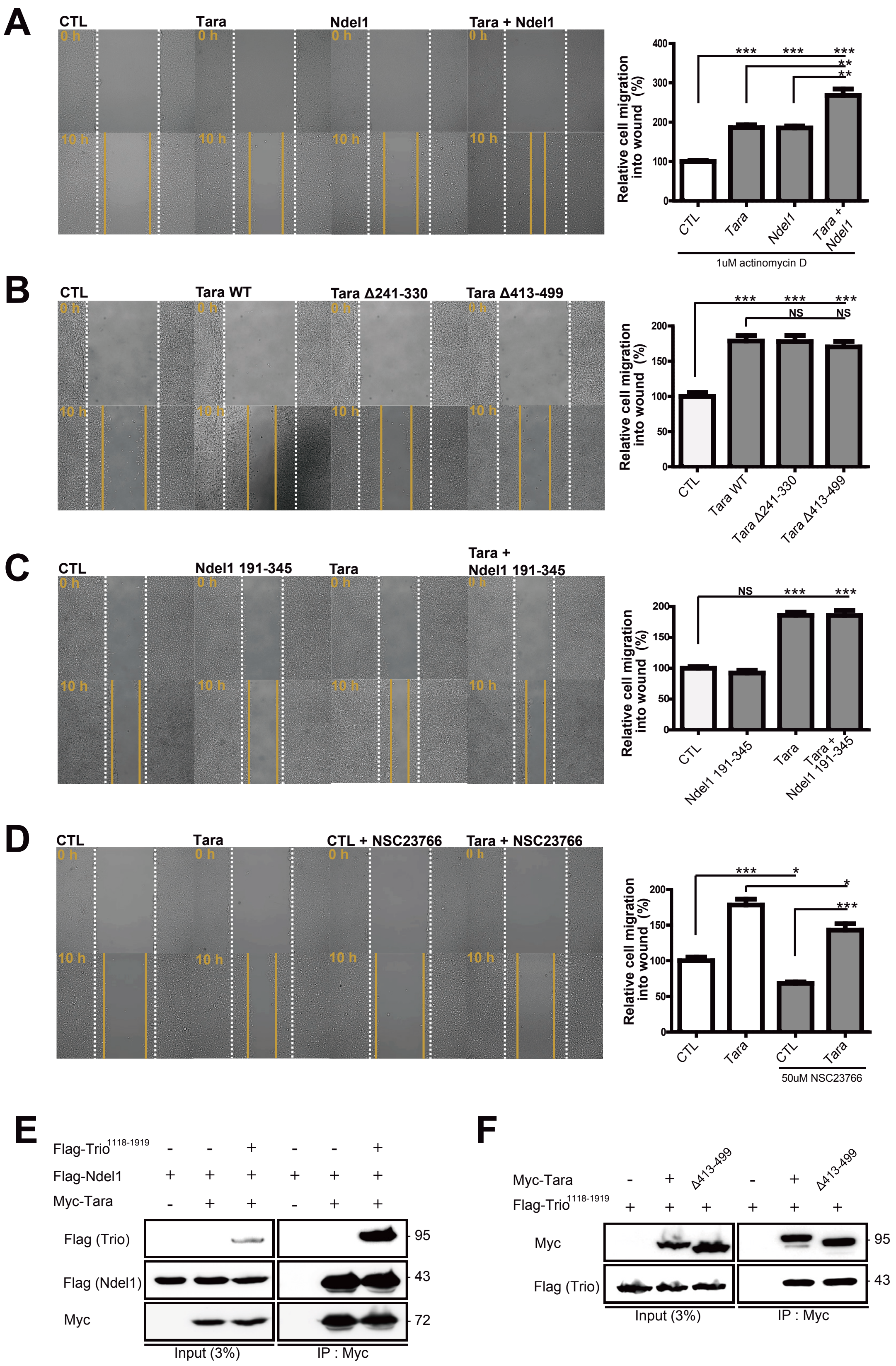
A
CTL
Tara
Ndel1
Tara + Ndel1
0 h
10 h
Relative cell migration into wound (%)
***
**
CTL
Tara
Ndel1
Tara + Ndel1
1uM actinomycin D
B
CTL
Tara WT
Tara Δ241-330
Tara Δ413-499
NS
CTL
Tara WT
Tara Δ241-330
Tara Δ413-499
C
CTL
Ndel1 191-345
Tara
Tara + Ndel1 191-345
D
CTL
Tara
CTL + NSC23766
Tara + NSC23766
*
50uM NSC23766
E
Flag-Trio1118-1919
Flag-Ndel1
Myc-Tara
Flag (Trio)
Flag (Ndel1)
Myc
95
43
72
Input (3%)
IP : Myc
F
Myc-Tara
Δ413-499
Flag-Trio1118-1919
Myc
Flag (Trio)
95
43
Input (3%)
IP : Myc

FIGURE S4

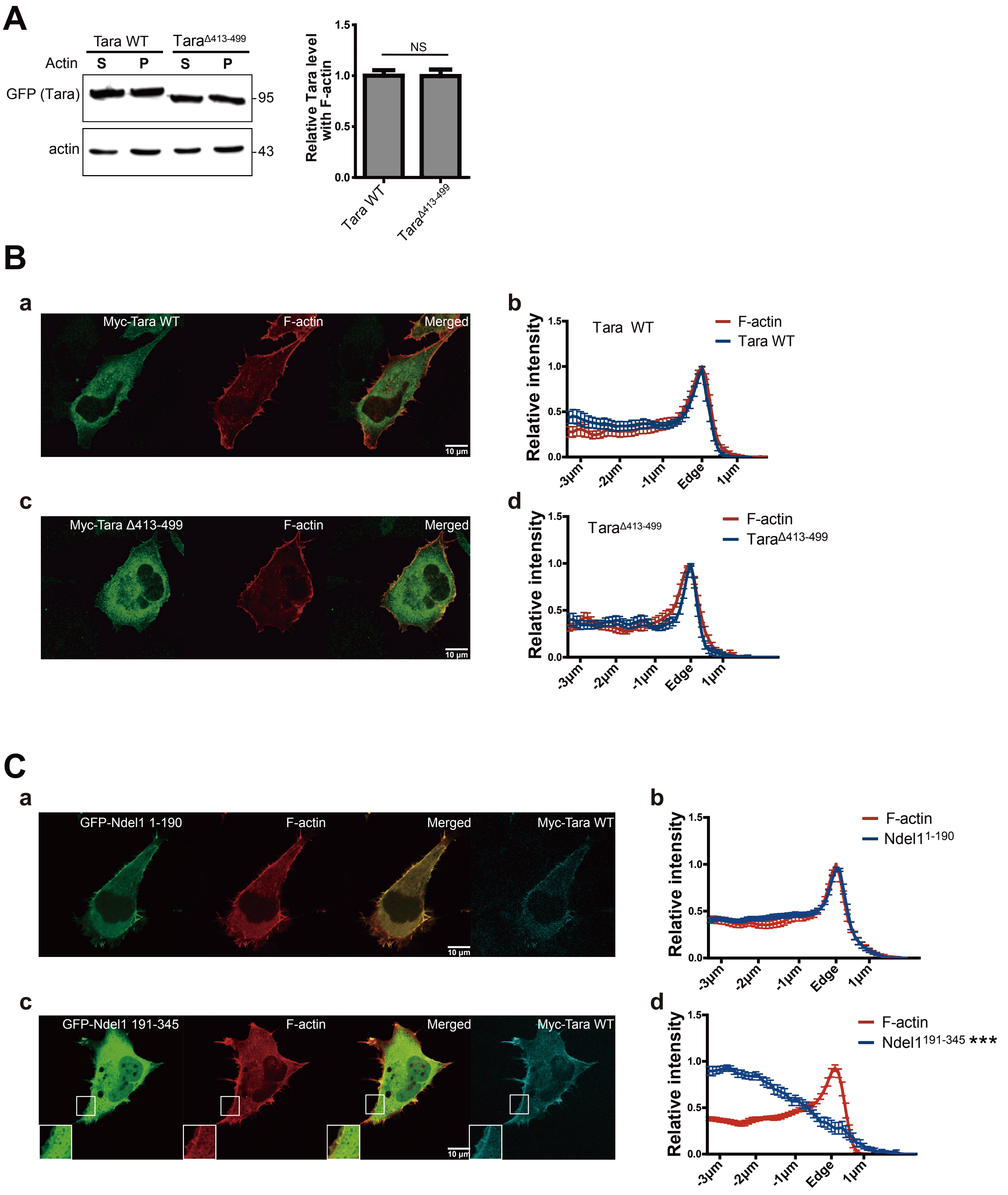

A
Tara WT
TaraΔ413-499
Actin
S
P
S
P
GFP (Tara)
95
actin
43
Relative Tara level with F-actin
NS
Tara WT
TaraΔ413-499
B
a
Myc-Tara WT
F-actin
Merged
10 μm
b
Relative intensity
Tara WT
F-actin
Tara WT
-3μm
-2μm
-1μm
Edge
1μm
c
Myc-Tara Δ413-499
F-actin
Merged
10 μm
d
TaraΔ413-499
F-actin
TaraΔ413-499
C
a
GFP-Ndel1 1-190
F-actin
Merged
Myc-Tara WT
10 μm
b
F-actin
Ndel11-190
c
GFP-Ndel1 191-345
F-actin
Merged
Myc-Tara WT
d
F-actin
Ndel1191-345 ***

FIGURE S5

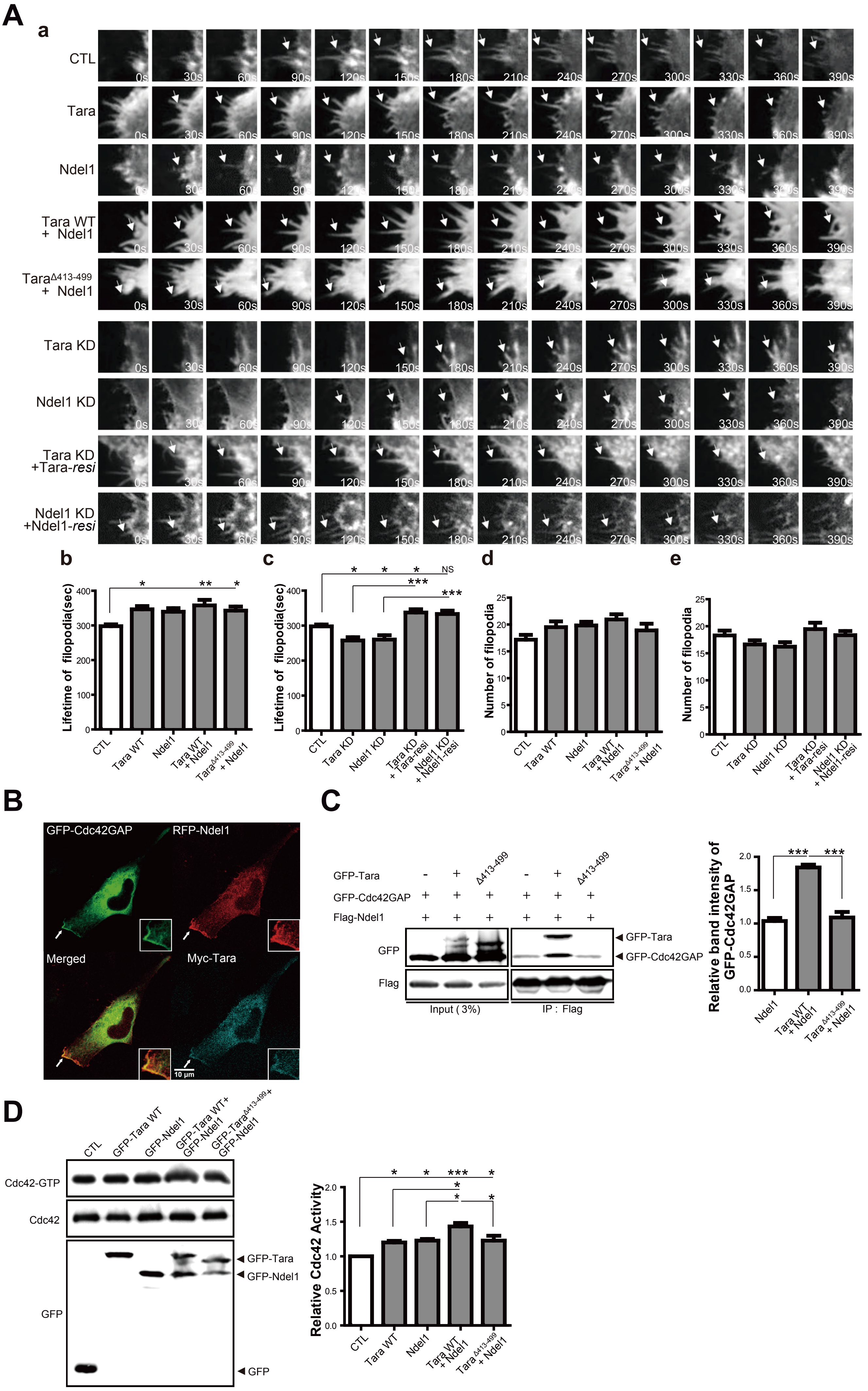
A
a
CTL
Tara
Ndel1
Tara WT + Ndel1
TaraΔ413-499 + Ndel1
Tara KD
Ndel1 KD
Tara KD +Tara-resi
Ndel1 KD +Ndel1-resi
0s 30s 60s 90s 120s 150s 180s 210s 240s 270s 300s 330s 360s 390s
b
Lifetime of filopodia(sec)
CTL
Tara WT
Ndel1
Tara WT + Ndel1
TaraΔ413-499 + Ndel1
c
Lifetime of filopodia(sec)
NS
CTL
Tara KD
Ndel1 KD
Tara KD + Tara-resi
Ndel1 KD + Ndel1-resi
d
Number of filopodia
e
Number of filopodia
B
GFP-Cdc42GAP
RFP-Ndel1
Merged
Myc-Tara
10 μm
C
GFP-Tara
GFP-Cdc42GAP
Flag-Ndel1
Δ413-499
GFP
Flag
Input (3%)
IP : Flag
◀ GFP-Tara
◀ GFP-Cdc42GAP
Relative band intensity of GFP-Cdc42GAP
Ndel1
Tara WT + Ndel1
TaraΔ413-499 + Ndel1
D
CTL
GFP-Tara WT
GFP-Ndel1
GFP-Tara WT+ GFP-Ndel1
GFP-TaraΔ413-499+ GFP-Ndel1
Cdc42-GTP
Cdc42
GFP
◀ GFP-Tara
◀ GFP-Ndel1
◀ GFP
Relative Cdc42 Activity
CTL
Tara WT
Ndel1
Tara WT + Ndel1
TaraΔ413-499 + Ndel1

FIGURE S6

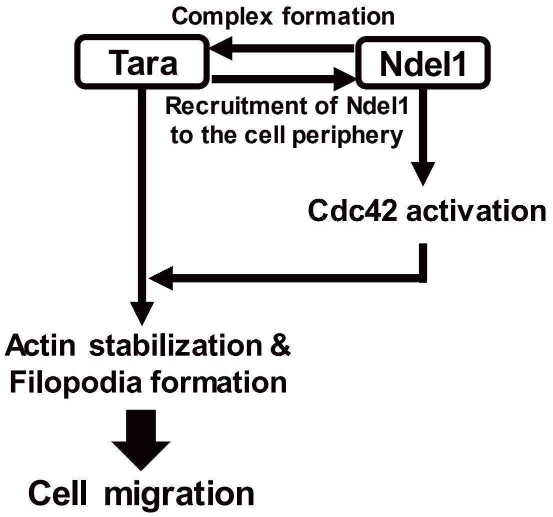
Complex formation
Tara
Ndel1
Recruitment of Ndel1
to the cell periphery
Cdc42 activation
Actin stabilization &
Filopodia formation
Cell migration
